# OA-GL21, a novel bioactive peptide from *Odorrana andersonii*, accelerated the healing of skin wounds

**DOI:** 10.1042/BSR20180215

**Published:** 2018-06-21

**Authors:** Wenxin Bian, Buliang Meng, Xiaojie Li, Siyuan Wang, Xiaoqing Cao, Naixin Liu, Meifeng Yang, Jing Tang, Ying Wang, Xinwang Yang

**Affiliations:** 1 Department of Anatomy and Histology and Embryology, Faculty of Basic Medical Science, Kunming Medical University, Kunming, Yunnan 650500, China; 2 Department of Biochemistry and Molecular Biology, Faculty of Basic Medical Science, Kunming Medical University, Kunming, Yunnan 650500, China; 3 Key Laboratory of Chemistry in Ethnic Medicine Resource, State Ethnic Affairs Commission and Ministry of Education, School of Ethnomedicine and Ethnopharmacy, Yunnan Minzu University, Kunming, Yunnan 650500, China

**Keywords:** Odorrana andersonii, skin secretions, wound healing promoting peptide

## Abstract

Nowadays, the number of chronic trauma cases caused by a variety of factors such as the world’s population-ageing and chronic diseases is increasing steadily, and thus effective treatment for chronic wounds has become a severe clinical challenge, which also burdens the patient both physically and financially. Therefore, it is urgent to develop new drugs to accelerate the healing of wounds. Bioactive peptides, which are relatively low cost, easy to produce, store and transport, have become an excellent choice. In this research, we identified a novel peptide OA-GL21, with an amino acid sequence of ‘GLLSGHYGRVVSTQSGHYGRG’, from the skin secretions of *Odorrana andersonii*. Our results showed that OA-GL21 exerted the ability to promote wound healing of human keratinocytes (HaCaT) and human fibroblasts in a dose- and time-denpendent manner. However, OA-GL21 had no significant effect on the proliferation of these two cells. Significantly, OA-GL21 showed obvious ability to promote wound healing in the full-thickness skin wound model in dose- and scar-free manners. Further studies showed that OA-GL21 had no direct antibacterial, hemolytic, and acute toxic activity; it had weak antioxidant activities but high stability. In conclusion, this research proved the promoting effects of OA-GL21 on cellular and animal wounds, and thus provided a new peptide template for the development of wound-repairing drugs.

## Introduction

Skin, as a physical barrier between the human body and the external environment, is responsible for many important physiological functions such as sweating, sensing heat and pain. Skin also protects tissues and organs inside the body from physical, mechanical, and chemical damages. At the same time, however, skin is the most vulnerable to injuries such as rupture, infection caused by pathogenic microorganisms, and other harmful factors [1,2]. Rapid repair after skin damage is an important precondition for homeostasis reconstruction in the body, which mainly consists of four stages: hemostasis, inflammatory response, cell proliferation, and tissue reconstruction [3]. This slow and complicated process can be easily affected by various factors, leading to a delay of the wound healing process or causing chronic wounds that are difficult to heal. Thus, the conditions enabling the invasion and reproduction of pathogenic microorganisms were created, which might lead to secondary sepsis, water electrolyte disorder, septic shock, multiple organ failure, and even death. All these cases cause serious physical and financial burden to patients [4,5]. Therefore, rapid healing of skin trauma is vital to the body.

In recent years, chronic skin trauma cases caused by challenges such as ageing and variety of chronic diseases are increasing steadily, leading to a major clinical challenge about chronic wound treating [6,7]. These cases inflict a severe economic burden on society; taking the United States as an example, this country spends more than $30 billion annually to deal with this issue. Since there are no satisfactory treatments and drugs for refractory chronic skin injury, it is urgent to develop a new type of more effective wound healing medicine [8]. Existing drugs for wound repair mainly include small molecular compounds derived from plants and proteins represented by epidermal growth factors (EGFs). However, all these drugs have some obvious shortages: the former is unstable in human body and has relatively low biological activity; the latter is costly and difficult to store, and can easily cause hypertrophic scars [9]. Therefore, it is very important to develop new wound healing drugs which can avoid these shortcomings [10]. Compared with small molecular compounds and growth factors, peptide molecules with high selectivity, high stability, and high activities, have caught the attention of scientists and companies working on innovative drug research and development. Ever since Lysine vasopressin was first discovered in the 1970s, great strides have been made in the development of polypeptide drugs, some of which, such as Copaxone, Exenatide, and Teriparatide Acetate, have become the best choice for patients with multiple sclerosis, type 2 diabetes, or osteoporosis, and have already obtained huge commercial success. Therefore, bioactive peptides have become a natural pharmaceutical resource bank in the field of drug development.

The surroundings of amphibians are quite complex. In the long-term process of natural selection, amphibians gradually evolved a unique and highly efficient skin peptide defense system, such as antibacterial peptides defending against pathogenic microorganisms and antioxidant peptides preventing the frogs from strong oxidative damage [11–14]. Previous studies have shown that amphibian skin can repair itself quickly after the damage, therefore, researchers hypothesized and confirmed that there is a necessary basic material in the skin to promote wound healing [15–17]. Amphibian skin secretions have a strong recovery activity, but very little is known about the specific effectors. So far, only several wound-healing-promoting peptides in amphibian skin secretions were reported [18–22]. Therefore, the studies of amphibian skin wound-healing-promoting peptides are still in their infancy and awaiting further research.

In amphibians, the odorous frog can secrete a variety of bioactive peptides [23]. *Odorrana andersonii* is a unique species of amphibians in China. It is mainly distributed in Yunnan, Guizhou, and other nearby provinces. It lives in an environment containing a large number of stones and jet streams. The bare skin *of O. andersonii* is vulnerable to damage, and as such, the skin damage must be healed quickly to ensure its survival. Based on this observation, we speculated that there are molecules that can promote wound healing in its skin secretions. In the present study, we found a new peptide, OA-GL21, from *O. andersonii*, which showed obvious wound-healing-promoting activity on both cell scratch and animal full-thickness skin wound models. These results provided a new template for the development of wound-healing-promoting drugs.

## Materials and methods

### Sample collection and animal care

Adult *O. andersonii* specimens (*n*=30) were collected from Baoshan city in Yunnan Province, China, and provided with mealworms in a 50 cm × 65 cm container. Several days were given to acclimate the environment before experiments. The skin secretions were collected according to the following procedures: stimulating the odorous frogs by an alternating current (6 V) using an electronic massager for 6–10 s, then washing the frog bodies with 25 mM Tris/HCl buffer (pH = 7.8), and collecting the skin secretions. The collected solutions were centrifuged at 5000 ***g*** for 30 min at 4°C and the supernatants were then collected, lyophilized, and stored at −80°C until use.

All animal care and handling procedures were conducted in accordance with the requirements of the Ethics Committee of Kunming Medical University (KMMU20180012).

### Purification procedure

**Figure 1:**
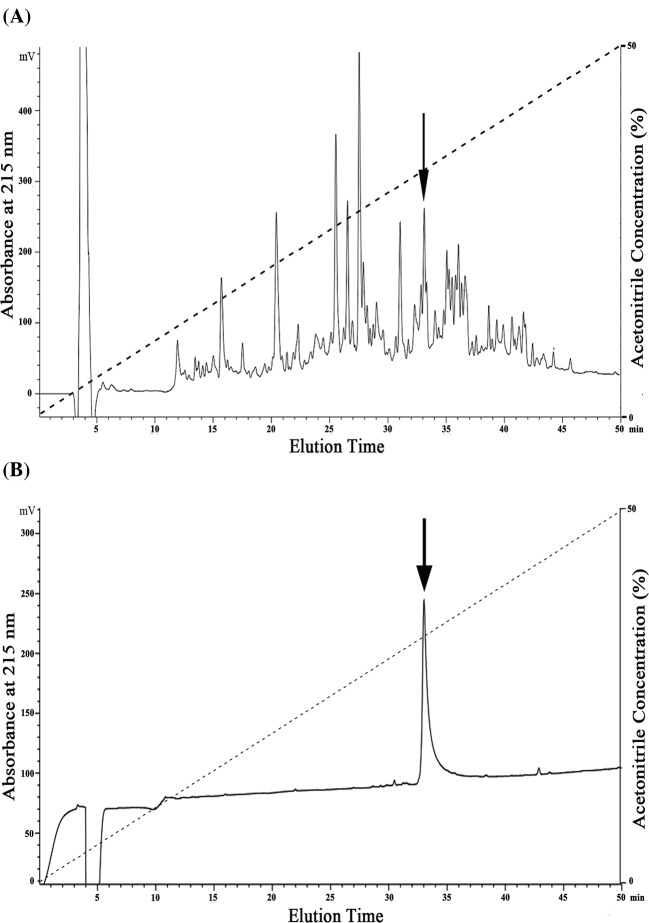
Peptide purification procedures from skin secretions of *O. andersonii* Skin secretions of *O. andersonii* were separated by a Sephadex G-75 column (shown as Figure 1A in our recent report [18]) and samples exhibiting wound healing activity were purified by a round of RP-HPLC. The sample with wound-healing-promoting activity was indicated by an arrow (**A**) and then further purified by another round of RP-HPLC by identical procedure with the first round of RP-HPLC. Finally, a peptide with wound-healing-promoting activity was obtained (indicated by an arrow in (**B**)) and is awaiting further research.

The purification procedure was performed as per previous research [18]. Briefly, after redissolving the lyophilized skin secretion samples with deionized water (500 μl, OD_280_ = 80) and applying it to a Sephadex G-75 (1.5 × 31 cm, superfine, GE Healthcare, Sweden) gel filtration column pre-equilibrated with 25 mM Tris/HCl buffer (pH = 7.8) containing 0.1 M NaCl, the elution was performed with the same buffer at a flow rate of 0.1 ml/min. Fractions were collected using an automatic fraction collector (BSA-30A, HuXi Company, Shanghai, China) every 10 min and monitored at 280 nm. After desalting the fractions by a reverse-phase HPLC (RP-HPLC) on a C4 column (4.6 × 250 mm, Elite, China), the collected samples were lyophilized, redissolved in 500 µl DMEM/F12 medium (BI, Israel), and their wound healing activities on human keratinocytes (HaCaT) cells were tested. Samples with cellular-level wound healing activity were merged together and then applied to a C18 RP-HPLC column (Hypersil BDS C18, 4.0 × 300 mm, Elite, China) pre-equilibrated with 0.1% (v/v) trifluoroacetic acid (TFA) in water. Elution was achieved by a linear gradient (0–50% acetonitrile (ACN) in 50 min, as shown in Figure 1A,B) of 0.1% (v/v) TFA in ACN under a flow rate of 1 ml/min and monitored at 215 nm at the same time. Peaks with cellular-level wound healing activity were collected and lyophilized for a second HPLC purification procedure using the same conditions as above.

### Determination of peptide primary structure

The average molecular mass of native OA-GL21 was determined by an AXIMA-CFRTM plus MALDI-TOF mass spectrometer (Shimadzu/Kratos, Manchester, U.K.) in linear mode with α-cyano-4-hydrorycinnamic acid as the matrix. Then the high-purity peptide was directly subjected to Edman degradation on a PPSQ-31A protein sequencer (Shimadzu, Japan) to obtain the complete amino acid sequence according to the manufacturer’s standard GFD protocols provided by the manufacturer, and data were analyzed using the provided software package.

### Cloning of cDNA encoding OA-GL21

An *O. andersonii* skin cDNA library was successfully constructed in our previous research [23], with the cDNA encoding mature peptide screened from this library. Briefly, the 5′-PCR primer (5′-CCAAA(G/C)ATGTTCACC(T/A)TGAAGAAA-3′) and 3′-PCR primer (5′-ATTCTAGAGGCCGAGGCGGCCGACATG-3′) were commercially synthesized and provided by the BGI Company (China). PrimeSTAR® HS DNA Polymerase (TaKaRa Biotechnology Co. Ltd., Dalian, China) was selected for PCR under the following conditions: 2 min at 94°C and then 25 cycles of 10 s at 92°C, 30 s at 50°C, and 40 s at 72°C. The PCR products were recovered using a DNA Gel Extraction Kit (Bioteke, China) and ligated into a pMD19-T Vector (TaKaRa Biotechnology Co. Ltd., Dalian, China). The PCR products were finally cloned into *Escherichia coli* DH5α and independent clones were chosen to carry out DNA sequencing on an Applied Biosystems DNA sequencer (ABI 3730XL, Foster City, CA, U.S.A.).

### Peptide synthesis

The mature OA-GL21 peptide (GLLSGHYGRVVSTQSGHYGRG) was commercially synthesized by Wuhan Bioyeargene Biotechnology Co., Ltd. (Wuhan, China). The synthesized OA-GL21 was analyzed by mass spectrometer, co-eluted with the natural OA-GL21, and confirmed to be identical with the native OA-GL21. The biological activities of the synthesized OA-GL21 were then tested.

### Hemolytic activity assay

Hemolytic activity was tested according to earlier studies [24]. In short, erythrocytes were washed thrice with normal saline or PBS first. Then the same volume of human erythrocytes (1% as the terminal concentration) and various doses of the OA-GL21 peptide were incubated together at 37°C in a water bath for 30 min. After centrifugation at 3000 ***g*** for 5 min at room temperature, absorbance of the supernatant was measured at 540 nm. Incubation with 0.1% Triton X-100 was carried out to determine maximum hemolysis. All the dissolvants used in this experiment must be normal saline or PBS for the fear that hemolysis might occur accidently.

### Acute toxicity assay

Acute toxicity was determined in two phases as described previously [25]. Lethal range of the OA-GL21 by acute toxicity test was determined at concentrations of 10, 25, and 50 µmol/kg by intraperitoneal (i.p.) injection in mice. Mice were observed for 24 h, and the mortalities, toxic effects, and changes in behavioral patterns were recorded.

### Cellular wound healing activity assay

Cellular wound healing was determined according to earlier research [19]. In short, HaCaT cells and human skin fibroblasts (HSFs) were cultured in DMEM/F12 medium (BI, Israel) with 10% FBS (BI, Israel), 100 units/ml of streptomycin and 100 units/ml of penicillin, and incubated in a humidified atmosphere of 5% CO_2_ at 37°C. Cell monolayer formation was achieved by culturing the HaCaT and HSF cells in 24-well plates (2.5 × 10^5^ cells/well) for 12–24 h. The cell monolayers were then wound by a yellow 200-µl pipette tip (Axygen, U.S.A.), and twice washed with PBS to remove any detached cells. Subsequently, DMEM/F12 medium serum free (500 µl) containing various concentrations of OA-GL21 (0.005, 0.05, 0.5, 5, and 50 µM) were respectively added to each well with or without mitomycin-C (10 µg/ml, Sigma–Aldrich, St Louis, MO, U.S.A.). Images of the wound healing monolayers were acquired using a Primovert microscope (Zeiss, Germany) at time intervals of 0, 6, 12, 18, and 24 h. Cell migration activity was expressed as the percentage of the gap relative to the total area of the cell-free region immediately after the scratch, named the healing rate of scarification, using ImageJ software (National Institutes of Health, Bethesda, MD, U.S.A.). For each plate, six randomly selected images were acquired. All experiments were independently carried out in triplicate.

### HaCaT and HSF cell proliferation assays

Cells were cultured same as above and then plated in 96-well plates (3000 HaCaT cells and 10000 HSF cells per well, 80 µl, respectively) incubated for 2 h to allow the cells to adhere to the well walls. Subsequently, 20 µl OA-GL21 (0.005, 0.05, 0.5, 5, and 50 µM) dissolved in DMEM/F12 (serum free) were added to each well, followed by a further 24 h of incubation. The same volume of DMEM/F12 (serum free) was used as a negative control. After incubation, the CellTiter 96® AQueous One Solution Assay (Promega, Madison, WI, U.S.A.) was used in accordance with the manufacturer’s instructions to test the effect of OA-GL21 on HSF and HaCaT cell proliferation [26].

### Animal wound healing assay

We obtained 20 adult male mice (22–25 g) from the Experimental Animal Center of Kunming Medical University. Keeping individually and providing water and laboratory feed normally, the animals were given several days to acclimate to the conditions before the experiments. Full-thickness skin wounds were then surgically created on the back of the mice [18]. The mice were anesthetized with i.p. injection using 100 ml solution containing 1% pentobarbital sodium solution (0.1 ml/20 g body weight). After preparing the back skin by shaving the back area and disinfecting with 75% ethanol swab, two 8 × 8 mm full-thickness excisional wounds were created on the back of each mouse. At the end of the surgical procedure, cages were placed near a heating apparatus until mice fully recovered from anesthesia. Randomly dividing the mice into four groups, the left-sided wounds of the first three groups were treated with 20 µl (1, 10, and 100 µg/ml, respectively) of OA-GL21, with the same volume of normal saline applicated on the right-side wounds as negative control; for the last group acted as possitive control, the left-sided wounds were treated with 20 µl (10 mg/ml) of Kangfuxin (KFX, the ethanol extract from the American cockroach, Inner Mongolia Jingxin Pharmaceutical Co. Ltd., China, Z15020805), a common trauma drug which can accelerate wound repair effectively. Similarly, the right-sided wounds were treated with the same volume of saline. All the wounds were treated twice daily. The wounds were imaged every 2 days [18].

Recording the closure of the mouse wounds with a D3000 digital camera (Nikon, Japan), the residual wound areas were estimated from the photographs by ImageJ software (NIH, U.S.A.). Mean values of successive tracings were computed as percentages of closure from the initial wound based on triplicate images using the following equation: healing rate of wound (%) = [R (3, 5, 7, 9)/R (1)] × 100, where R (1) and R (3, 5, 7, 9) note the remaining wound area at the same day of operation and post-operative days 3, 5, 7, and 9, respectively. Wound-healing curves were constructed by GraphPad Prism software (v.5).

### Tissue preparation and histological analysis

Mice wound tissue samples were taken at post-operative day 9 and fixed over 24 h in 4% paraformaldehyde, suffered by dehydration and transparent processing, then embedded in paraffin. For histological analysis, tissue samples were sectioned into 5-µm thickness slices and all sections were deparaffinized, rehydrated, and stained with Hematoxylin and Eosin (H&E). All slices ware imaged by a Primovert microscope (Zeiss, Germany) and used to observe the regeneration of epidermal and granulation. The thickness of neoepidermis and granulation was measured by ImageJ software (NIH, U.S.A.).

### Antimicrobial activity assay

In this research, the antimicrobial activity of OA-GL21 was tested by the high sensitive plate method [12]. Here, Gram-positive bacterial strains: *Staphylococcus epidermidis* (ATCC 12228), *Staphylococcus haemolyticus* (ATCC 29970), *Bacillus subtilis* (ATCC 19659); Gram-negative bacterial strains: *E. coli* (ATCC 25922), *Aeromonas hydrophila* (ATCC 49140), *Streptococcus iniae* (ATCC 29177), *Vibrio splendidus* (ATCC 33869); and fungal strain: *Candida albicans* (ATCC 14053), were obtained from the Kunming Medical University. After growing the microbes in LB broth to OD_600_ = 0.8, a 10-µl aliquot of the bacteria was added to 10 ml fresh LB broth with 1% Type I agar (Sigma–Aldrich, St Louis, MO, U.S.A.) and poured over a 90 mm Petri dish. After the agar hardened, a small hole was made and a 7-µl aliquot of OA-GL21 (1 mM) was added to the hole, followed by overnight incubation at 37°C. A clear zone would form on the surface of the agar representing inhibition of bacterial growth, while the sample had antimicrobial activity. In this experiment, ampicillin (1 mg/ml) was used as a positive control.

### Antioxidant activity assay

We used free radical scavenging assay *in vitro* to detect the antioxidant activity of OA-GL21. A2,2′-azino-bis(3-ethylbenzothiazoline-6-sulphonic acid) (ABTS) scavenging test was performed according to previously described procedures [13], with some modifications. Briefly, a stock solution of ABTS radical (Sigma–Aldrich, St Louis, MO, U.S.A.) was prepared by incubating 2.8 mM potassium persulphate (Sigma–Aldrich, St Louis, MO, U.S.A.) with 7 mM ABTS in water for at least 6 h in the dark, after which it was used immediately. The stock solution was diluted 50-fold with deionized water. Different concentrations of OA-GL21 dissolved in water were added, with the same volume of solvent being used as the negative control. Vitamin C (10 µM) dissolved in H_2_O was used as the positive control. The reaction was kept from light for 30 min. The decrease in absorbance at 415 nm indicated the antioxidant activity of the samples. The ABTS^+^ scavenging rate (%) was calculated by (A_blank_ – A_sample_) ×100/A_blank_.

A2,2-diphenyl-1-picrylhydrazyl (DPPH) scavenging test was also performed as previously described [13]. In short, the assay mixture contained 190 µl of 5 × 10^−5^ M DPPH radical (Sigma–Aldrich, St Louis, MO, U.S.A.) dissolved in methanol and 10 µl sample solution. The mixture was then incubated in a 96-well plates for 30 min keeping in dark at room temperature. The absorbance was read against a blank at 517 nm. The DPPH^+^ scavenging rate (%) was calculated by (A_blank_ – A_sample_) × 100/A_blank_ (10 µM vitamin C as positive control).

### Stability analysis of OA-GL21

The stability of OA-GL21 at 4 and 37°C was checked. Briefly, OA-GL21 (10 µg/ml dissolved in deionized water) was incubated at 4 and 37°C for different days, samples were taken at the selected time points and then centrifuged at 12000 ***g*** for 20 min, and then supernatants were analyzed by C18 RP-HPLC (Hypersil BDS C18, 4.0 × 300 mm, Elite, China). The intacted OA-GL21 levels were quantitated from peak (corresponding to the elution time of OA-GL21) absorbance at 215 nm.

## Result

### Peptide purification

Skin secretions of *O. andersonii* were collected every 10 min through gel filtration, and three main fractions were obtained in our previous research (shown by a black arrow in Figure 1A in our recent report [18]). The peak indicated by an arrow was purified by RP-HPLC process, and we received more than 30 peaks (Figure 1A in this manuscript, corresponding to the Figure 1B in our recent report [18], but with different indicating arrows). For every peak, the cellular level wound-healing-promoting activity was tested, and we observed that one of them showed the expected activity (shown by a black arrow in Figure 1A). This peak was further purified by another round of RP-HPLC, and finally we obtained a sample with ideal peak shape (indicated by an arrow in Figure 1B). Then the ability to promote HaCaT cell scratch repair and its primary structure was determined and confirmed.

### Primary structure of OA-GL21

**Figure 2:**
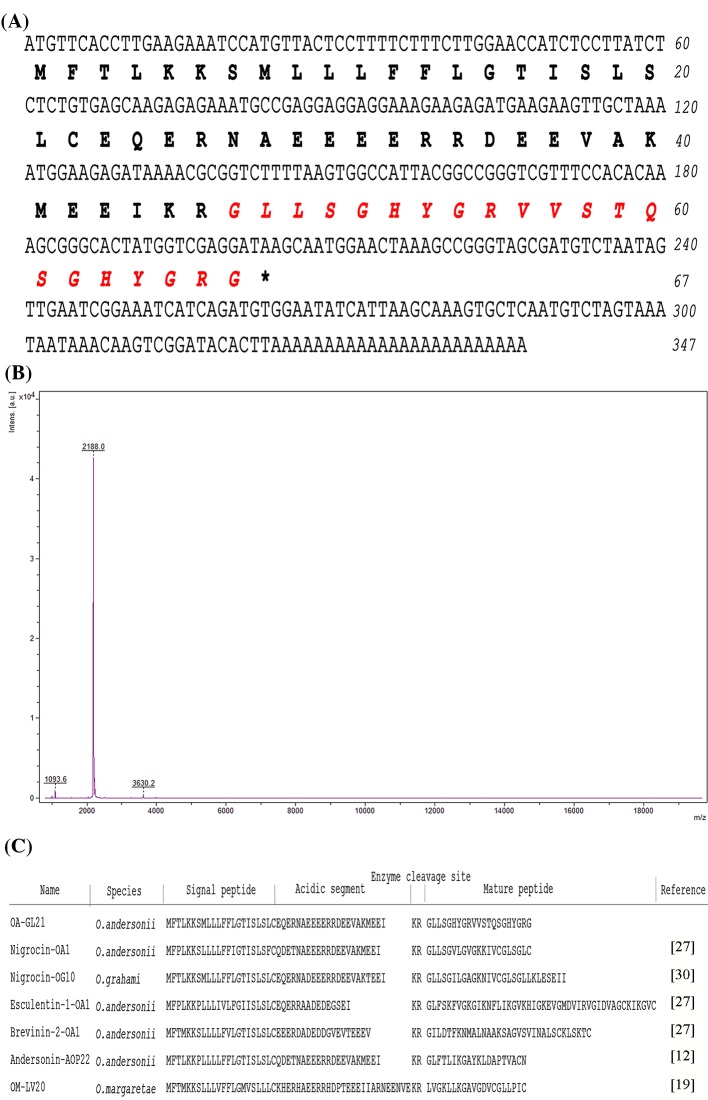
Primary structure of OA-GL21 Sequence of OA-GL21. The complete sequence of mature OA-GL21 is ‘GLLSGHYGRVVSTQSGHYGRG’, which has 21-amino acid residues in length (shown in red) and produced by post-translational modification of a 67-residue prepropeptide. (**A**) Observed molecular mass of native OA-GL21 and purity of peak, indicated by an arrow in Figure 1B. (**B**) The sequence alignment of OA-GL21 with other bioactive peptides from amphibian skins. (**C**) The sequence alignment of OA-GL21 with other bioactive peptides from amphibian skins.

The sample obtained from the second RP-HPLC (indicated by an arrow in Figure 1B) was analyzed by MS, revealing its observed molecular mass to be 2188.0 Da (Figure 2B). Comparing the result of MS analysis with secondary HPLC chromatographic results (Figure 1B), we speculated that this sample possessed an ideal purity of >90%, and was mainly composed of a peptide with a molecular mass of 2188.0 Da. In order to confirm its primary structure, we determined the amino acid sequence of this peptide by Edman sequencing, and got a sequence ‘GLLSGHYGRVVSTQSGHYGRG’ (Figure 2A) from N-terminus to C-terminus, and then successfully screened the cDNA encoding this peptide from an *O. andersonii* skin cDNA library. The 21-residue mature peptide was produced by post-translational processing of a 67-residue prepropeptide (Figure 2A, GenBank accession number: MG860487). The theoretical molecular weight of this peptide was 2188.39 Da (calculated at http://web.expasy.org/compute_pi/), which matched well with the observed molecular weight, indicating that there were no post-translational modifications. As shown in Figure 2C, we blasted this sequence in NCBI databases and found that this peptide showed sequence similarity with other amphibian bioactive peptides, such as Nigrocin family [23,27]; however, we considered OA-GL21 as a novel family due to the observation that there was no intramolecular disulphide bridge or free cysteine and thus this peptide was named as OA-GL21 accordingly (OA: species name abbreviation, GL: two initial amino acids, 21: peptide length).

### OA-GL21 showed no hemolytic activity against human blood cells and no acute toxicity against mice

**Figure 3:**
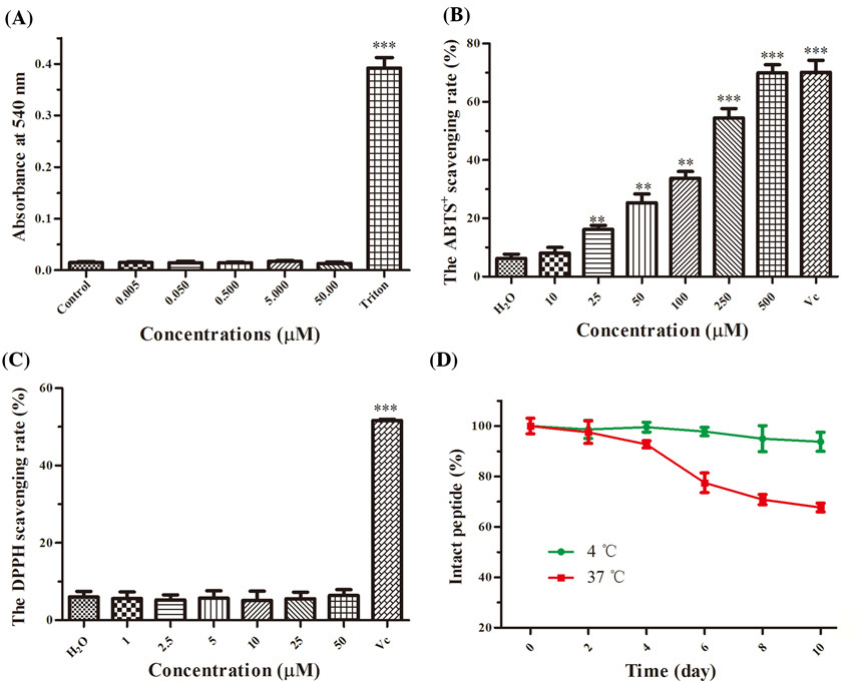
The hemolytic, antioxidant activities and stability of OA-GL21 (**A**) At the same concentrations of cellular wound healing activity assay, OA-GL21 showed no hemolytic activity against human blood cells. (**B**) OA-GL21 showed scavenging activity against free radical ABTS^+^ in a dose-dependent manner when at the concentrations ranging from 10 to 500 µM. (**C**) OA-GL21 showed no obvious scavenging activity against free radical DPPH. (**D**) OA-GL21 was highly stable at 4°C and began to decrease after 4 days but also maintained above 60% within 10 days at a higher temperature (37°C). Data are means ± S.E.M. of six independent experiments. **P<0.01 and ***P<0.0001 indicate significant difference from the control or H_2_O (Student’s *t*tests).

In view of many bioactive peptides having direct damaging effects on normal cells [28,29], we tested whether OA-GL21 had hemolytic activity and acute toxicity before further studies. The results showed that even at the maximum concentration of 50 µM, OA-GL21 had no obvious hemolytic activity (Figure 3A). Also in the acute toxicity test, no lethal effect was observed after a single i.p. injection of OA-GL21 (10, 25, and 50 µmol/kg, respectively) after 24 h (Table 1), and abnormal behaviors such as tremor, retardation, irritability, piloerection, and stiffness of tail were also not observed.

**Table 1:** Mouse mortality at different inoculation doses of OA-GL21

Group	Dosage (μmol/kg)	Number of mice		Mortality rate (%)
		Male	female	
Negative control (saline injection)	50	3	3	0
Experimental group				
Group 1	10	3	3	0
Group 2	25	3	3	0
Group 3	50	3	3	0

### OA-GL21 promoted HaCaT cell scratch healing

**Figure 4:**
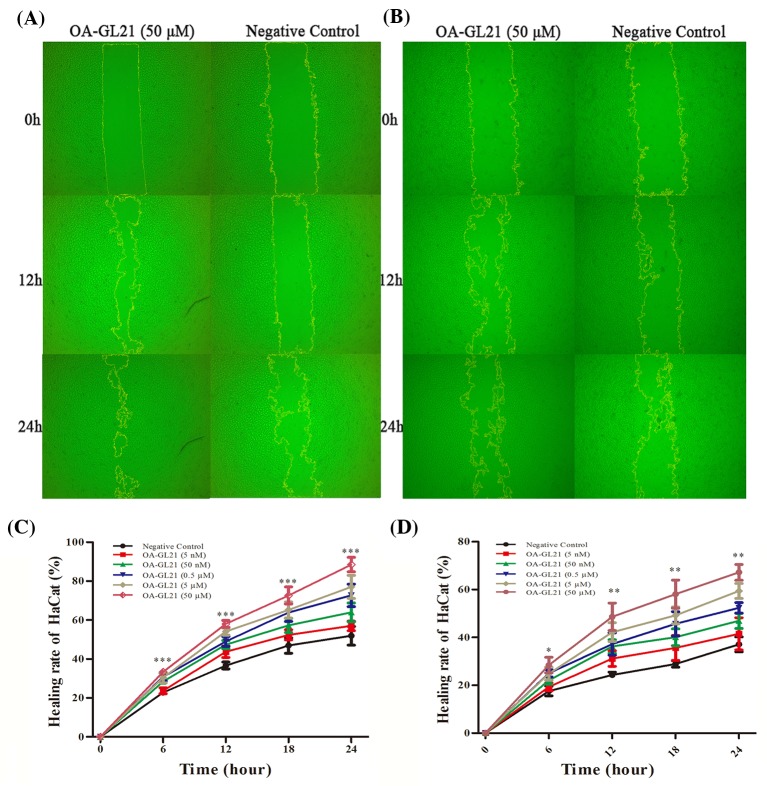
Effect of OA-GL21 on the healing rate of HaCaT cell scratch (**A**) OA-GL21 (50 µM) accelerated HaCaT cell scratch healing significantly. (**B**) OA-GL21 (50 µM) accelerated HaCaT cell scratch healing while proliferation was inhibited by mitomycin-C. (**C**) OA-GL21 showed time- and dose-dependent HaCaT cell wound-healing-promoting activity. (**D**) OA-GL21 showed time- and dose-dependent HaCaT cell wound-healing-promoting activity while proliferation was inhibited by mitomycin-C. Data are means ± S.E.M. of six independent experiments. *P<0.05, **P<0.01, and ***P<0.0001 indicate significant difference from the negative control (Student’s *t*tests).

The early migration of keratinocytes is very important in the healing process of skin wounds. Keratinocytes migrate to the wound area in the early stage of wound healing and form a new lingual epidermis covering the wound, which is conducive to the quick repair of the wound [3]. In this study, the effects of OA-GL21 on cell migration were observed *in vitro*. The migration rate of keratinocytes is the invasion efficiency of single-layer cell in 0–24 h with the premise of OA-GL21. As shown in Figure 4A, the background healing rate of HaCaT cells in the 12 and 24 h after the scratch was approximately 35 and 50%, respectively. After adding OA-GL21 (50 µM), the healing rate of 12 and 24 h was increased significantly to about 55 and 85%, respectively. Next, we studied the effects of OA-GL21 on the HaCaT cell migration with 0.005, 0.05, 0.5, 5, and 50 µM concentrations. As shown in Figure 4C, depending on time and dosage, OA-GL21 significantly promoted HaCaT cell scratch healing rates. According to the data analysis, at 24 h, OA-GL21, ranging from 0.5 to 50 µM, could promote the repair of HaCaT cells to more than 65%. After considering that during the process of cellular wound healing, both cell proliferation and migration have important effects on promoting the repair process, we used mitomycin-C (10 µg/ml) to suppress the cell proliferation, and observed that OA-GL21 could also accelerate the healing of HaCaT scratch; although the background healing rate was slower than that without mitomycin-C (Figure 4B,D). Compared with the negative control, the healing rate of HaCaT cells significantly increased from 35% to approximately 65% at 24 h after the addition of 50 µM OA-GL21. Overall, at the concentration from 0.005 to 50 µM, the results of these experiments showed OA-GL21 promoted the healing of HaCaT cell scratch in dose- and time-dependent manner, mainly by accelerating the cell migration.

### OA-GL21-promoted HSF cell scratch healing

**Figure 5:**
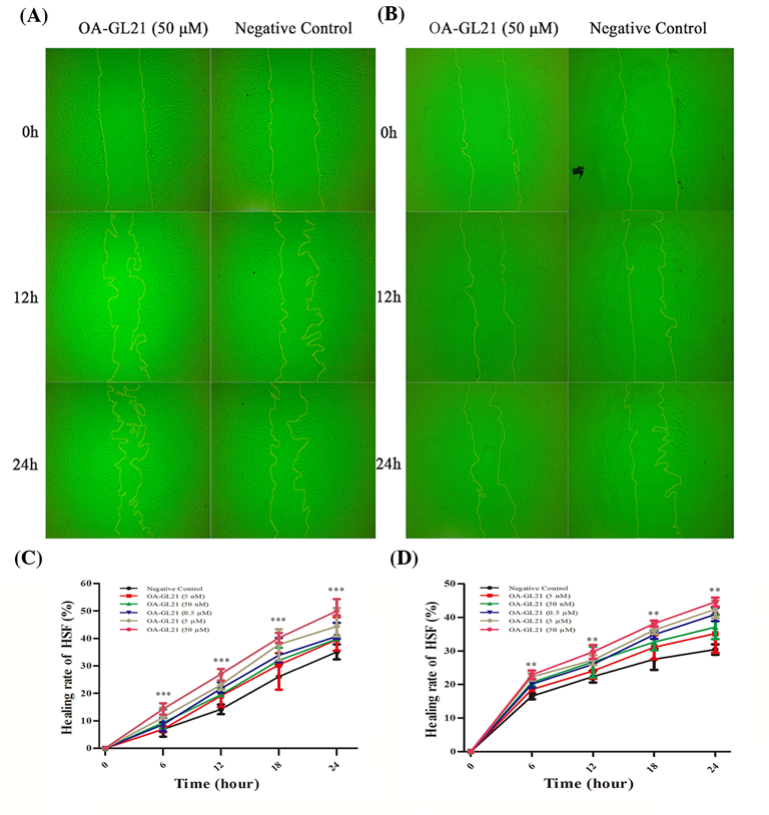
Effect of OA-GL21 on healing rate of HSF cell scratch (**A**) OA-GL21 (50 µM) accelerated HSF cell scratch healing significantly. (**B**) OA-GL21 (50 µM) accelerated HSF cell scratch healing while proliferation was inhibited by mitomycin-C. (**C**) OA-GL21 showed obviously time- and dose-dependent HSF cell wound-healing-promoting activity from concentration 0.5 to 50 µM. (**D**) OA-GL21 showed time- and dose-dependent HSF cell wound-healing-promoting activity while proliferation was inhibited by mitomycin-C. Data are means ± S.E.M. of six independent experiments. **P<0.01 and ***P<0.0001 indicate significant difference from the negative control (Student’s *t*tests).

In addition to keratinocytes, the migration of fibroblasts also plays an important role in wound healing process [3]. The effects of OA-GL21 on HSF cell migration was tested same as that of HaCaT cells. OA-GL21 showed a significant time-dependent activity at the concentration of 50 µM, as displayed in Figure 5A,C. Compared with the negative control, the healing rate of the HSF cell scratch for 24 h increased from 33 to 48% while cultured together with OA-GL21 (50 µM). In addition, the contribution of OA-GL21 to HSF cell migration increased with the increase in concentrations (from 0.5 to 50 µM). Similarly with HaCat cells, when using mitomycin-C (10 µg/ml) to inhibit the HSF cell proliferation, the background healing rate showed a degree of decrease (Figure 5C,D). OA-GL21 could also induce healing time and dose dependency. As a result, similar to that on HaCaT cells, the effect of OA-GL21 on HSF cells wound healing was dose- and time-dependent, mainly by promoting cell migration rather than inducing cell proliferation.

### OA-GL21 did not induce proliferation of HaCaT and HSF cells

**Figure 6:**
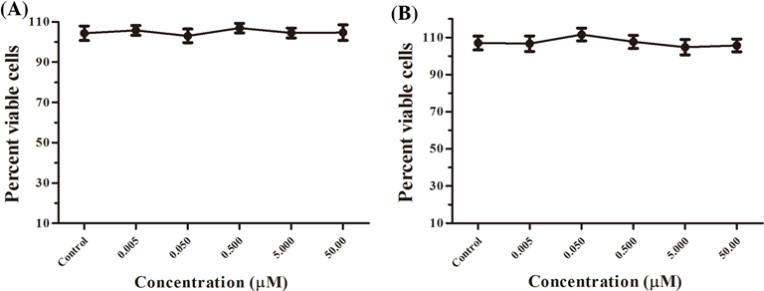
Effects of OA-GL21 on the proliferation of HaCaT and HSF cells (**A**) OA-GL21 showed no effect on the proliferation of HaCaT cells. (**B**) OA-GL21 showed no effect on the proliferation of HSF cells (*P*>0.05, Student’s *t*tests; ‘Control’ meant Negative control).

As mentioned above, in addition to cell migration, the proliferation of keratinocytes and fibroblasts is also important for wound healing [3]. In this research, the effects of OA-GL21 on cell proliferation were studied by cell proliferation assay *in vitro*. The results showed that at the same concentrations as the scratch test, OA-GL21 had no obvious effect on the proliferation of HaCaT and HSF cells (Figure 6A,B).

### OA-GL21 promoted the healing of full-thickness wounds in mice

**Figure 7:**
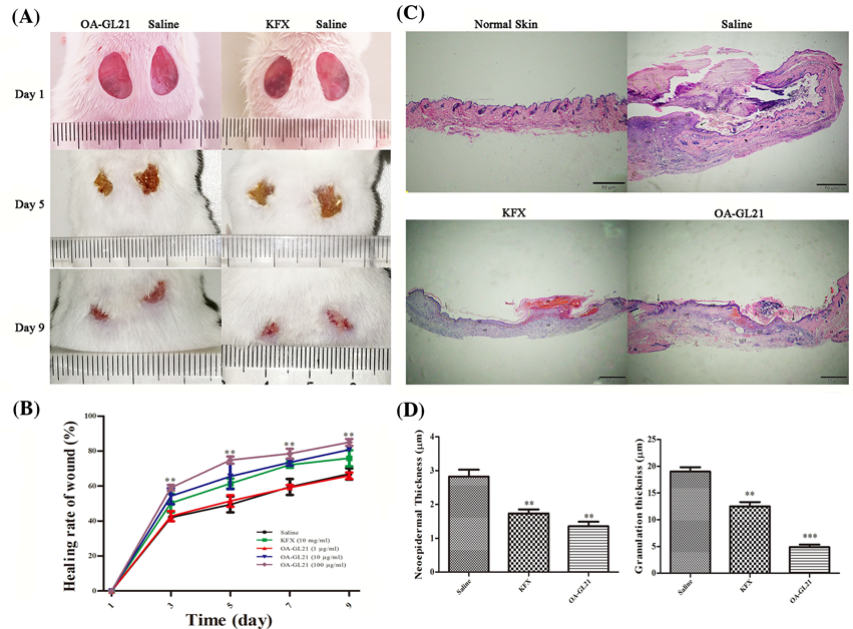
Effects of OA-GL21 on mice full-thickness skin wounds and histological analysis (**A**) Macroscopic view representative mice wounds were taken on post-operative days 1, 5, and 9. (**B**) Wound closure was assessed by morphometrical analysis of wound areas. Wound residual areas were determined (*n*=5). OA-GL21 (100 µg/ml) accelerated mice wound healing obviously. (**C**) Histological analysis of mice normal skin, saline-, KFX- (10 mg/ml), and OA-GL21- (100 µg/ml) treated full-thickness wound tissues stained with H&E. (E, epidermal; ES, escar; NE, neoepidermal; GT, granulation tissue; D, dermis; HF, hair follicle; M, muscle layer; LCT, loose connective tissue. Bars indicate 50 µm, arrow, wound edge.) (**D**) Thickness of neoepidermis and granulation tissues was measured and quantitated, and OA-GL21 induced thinner neoepidermis and granulation tissue than both saline and KFX group. **P<0.01 and ***P<0.0001 indicate significant difference from the negative control (Student’s *t*tests).

Given that OA-GL21 demonstrated obvious extracorporeal cell wound-healing-promoting activity, we hypothesized that it might also promote wound repair *in vivo.* In the following experiments, we explored the effects of local external use of OA-GL21 on the full-thickness wound model. We successfully established the full-thickness wound model in mice and the wounds were applied topically by different concentrations (1, 10, and 100 µg/ml) of OA-GL21 (20 µl) twice a day, with KFX (10 mg/ml, 20 µl, a commercially available wound healing promoting drug in China) as the positive control, and physiological saline (20 µl) as negative control. The trauma images were retained at the different days of post-surgery (day 1, 3, 5, 7, and 9), and the wound areas were calculated by the ImageJ software. Compared with the negative control, OA-GL21 (100 µg/ml) had a significant accelerating effect on the skin wound repair of mice (Figure 7A), while OA-GL21 (10 µg/ml) and KFX (10 mg/ml) showed similar recovery activity (Figure 7B), indicating that the activity of this peptide is approximately 1000 times stronger than KFX. In addition, when the concentration of OA-GL21 was 1 µg/ml, there was no obvious effect on mouse skin wound repair (Figure 7B). In summary, at the animal level, OA-GL21 also had a significant dose-dependent wound-healing-accelerating activity from 10 to 100 µg/ml. Besides, after local application of OA-GL21, the mice did not find any adverse reactions to weight, general health status, or behavior (data not shown).

Histological analysis revealed that compared with saline group, mice topically treated with OA-GL21 (100 µg/ml) showed significant increase in the regeneration of epidermis and dermis; moreover, thinner neoepidermis and better granulation tissue contraction were also observed (Figure 7C,D). At day 9, ideal re-epithelialization and well-formed granulation tissue were observed in both OA-GL21-treated and KFX-treated (10 mg/ml) groups (Figure 7C). The newly formed thinner epidermis covered approximately all the wound area and granulation tissue was well-structured. On the contrary, mice treated with saline had thicker hyperproliferative wound epidermis, the new ligule epidermal layer had not yet covered the wound area, and newly formed granulation tissue was constructed disorderly. The thickness of neoepidermis and granulation tissue were also measured and quantitated (Figure 7D). Both KFX and OA-GL21 led to the appearance of obvious thinner epidermis and granulation tissues than saline group, and OA-GL21 showed more potent activities than KFX. These results indicated that OA-GL21 could accelerate the healing of skin wounds effectively at a scarless or less-scar manner.

In summary, OA-GL21 significantly accelerated the healing of mice skin wounds in dose- and time-dependent manner; moreover, OA-GL21 also induced much less formation of scar.

### Antimicrobial and antioxidant activity

Considering the sequence of OA-GL21 was similar to some known bioactive peptides, which exerted direct microbe-killing or antioxidant abilities, we also tested the antimicrobial activity and antioxidant activity of OA-GL21. As listed in Table 2, when at the maximum concentration of 1 mM, OA-GL21 showed no direct bactericidal and fungicidal effects on the eight microbe strains. Meanwhile, we also tested its antioxidant activity and found that at the same concentration (50 µM), OA-GL21 had no obvious scavenging activities against free radical DPPH, but did show weak ability to scavenge ABTS^+^ (Figure 3B,C). At the maximum concentration (500 µM), OA-GL21 could scavenge approximately 70% ABTS^+^. All these results demonstrated the weak antioxidant activities of OA-GL21.

**Table 2:** Antimicrobial activity of OA-GL21

Microorganisms	Antimicrobial activity		
	AMP (1 mg/ml)	Saline	OA-GL21 (1 mM)
**Gram-positive bacteria**			
*S. epidermidis* (ATCC 12228)	+	*-*	*-*
*S. haemolyticus* (ATCC 29970)	+	*-*	*-*
*B. subtilis* (ATCC 19659)	+	*-*	*-*
**Gram-negative bacteria**			
*E. coli* (ATCC 25922)	+	-	-
*A. hydrophila* (ATCC 49140)	-	-	-
*S. iniae* (ATCC 29177)	+	-	-
*V. splendidus* (ATCC 33869)	+	-	-
**Fungal strains**			
*C. albicans* (ATCC 14053)	+	-	-

‘+’ indicates antimicrobial activity. ‘-’ indicates no antimicrobial activity.

### Stability of OA-GL21

Considering the potential possibility of OA-GL21 to be developed as a novel wound healing promoting drug candidate, we further checked the stability of OA-GL21 at 4 and 37°C. As illustrated in Figure 3D, the intact OA-GL21 showed no obvious decrease even at day 10, indicating that OA-GL21 was constantly stable at 4°C. On the contrary, when at 37°C, OA-GL21 began to decrease after 4 days but also maintained above 60% within 10 days. These results indicated that OA-GL21 had a high stability at low temperature (4°C), but when stored at higher temperature (37°C), OA-GL21 also had the chance to degrade significantly.

## Discussion

After long-term natural selection, the amphibians have evolved a unique and highly efficient skin peptide defense system, which is mainly composed of antibacterial peptides, antioxidant peptides, lectin, and protease inhibitors [30–33]. With new structure and functional diversity, amphibian skin peptides have a lot of clinical research values and many possible applications. Therefore, amphibians are regarded as a treasure trove for the development of new natural medicines [34]. Many studies have shown that amphibian skin can repair itself quickly after traumatic events, and the skin secretions of amphibians can also significantly accelerate the wound-healing process [15–17]. So far, however, only few related bioactive peptides have been identified [18–22]. The skin of the odorous frog can secrete a variety of bioactive peptides [23], which provides an infinite amount of possibilities for the discovery of new bioactive peptides. The purpose of the present study was to identify new type of wound healing peptides from the skin secretions of *O. andersonii*.

We purified a peptide with cellular level wound-healing-promoting activity from the skin secretions of *O. andersonii* (indicated by an arrow in Figure 1B). By the process of Edman sequencing and cDNA cloning, the mature sequence of this peptide was determined to be ‘GLLSGHYGRVVSTQSGHYGRG’ (Figure 2A), and no post-translational modifications were observed. After a BLASTp in the NCBI database, no same sequence was retrieved, so this peptide was believed completely new and named OA-GL21 accordingly (Figure 2C). As cDNA cloning results show, OA-GL21 was produced by post-translational processing of a 67-amino-acid residue (Figure 2A). The prepropeptide of OA-GL21 kept the same highly conservative template with other amphibian skin bioactive peptides, consisting of an N-terminal hydrophobic signal peptide, an acidic segment, and mature peptides at the C-terminal. Its enzyme-cutting site was the most common typical site, ‘lysine–arginine’ [23,27]. Notably, OA-GL21 was structurally different from most of other wound healing peptides with an intramolecular disulphide bridge, including OM-LV20, Cathelicidin-OA1, and tiger 17 [18,19,22]; besides, another prohealing peptide, CW49, contains one free cysteine [21]. Based on our knowledge, this was the first discovery of prohealing amphibian skin peptide without any disulphide bridge or free cysteine residue. Given that many bioactive peptides have direct damaging effects on normal cells [28,29], OA-GL21 was tested whether it had hemolytic activity and acute toxicity before further studies. Fortunately, OA-GL21 did not show the acute toxicity of human erythrocyte hemolysis and animal level (Figure 3A and Table 1).

In skin wound healing process, keratinocytes and fibroblasts played an extremely important role. In addition to forming a new tongue, epithelial wound cover at the beginning of the trauma to speed up the repair, they are mainly involved in proliferation period of repair [3]. As shown in Figures 4 and 5, OA-GL21 promoted the healing of cell scratch at dose- and time-dependent manner. Cell proliferation is also an important stage in the process of wound repair. However, OA-GL21 showed no effect on the proliferation of HaCaT or HSF cells (Figure 6). At the same time, while adding mitomycin-C to inhibit cell proliferation, although the background healing rate decreased slightly, OA-GL21 still promoted wound healing of HaCaT and HSF cells significantly (Figures 4B,D and 5B,D). These results suggested that OA-GL21 induced HaCaT and HSF cell wound healing by influencing the cell migration, rather than through the induction of proliferation, which was quite different with many of other prohealing peptides inducing both the migration and the proliferation of cells [18,19]. However, this might imply the less-scar or scarless healing of OA-GL21 on skin wounds. In summary, the detailed molecular mechanism of OA-GL21 exerting its accelerating effects on skin wounds was not sufficiently explored in this research and is awaiting future research.

Notably, OA-GL21 demonstrated significant effect on the repairing of the full-thickness wound model (Figure 7). As shown in Figure 7B, OA-GL21 accelerated the mice wound healing more obviously than saline-treated group and KFX-treated group. Moreover, the activity of OA-GL21 is approximately 1000 times stronger than KFX. Results of histological analysis showed that OA-GL21 accelerated mice wound healing by good re-epithelialization and well-formed granulation tissue (Figure 7C,D). These results meant that OA-GL21 could not only accelerate the healing process of the skin wounds and avoid the chronic wounds, but also reduce the formation of scar, and form a scarless or less-scar repair, which had obvious advantages over the existing wound healing drugs (small molecular compounds and growth factors).

Because the overall structure of OA-GL21 and other bioactive peptides from amphibian skin had been found similar (Figure 2C), we tested other relative activities of OA-GL21 according to these bioactive peptides. OA-GL21 showed no direct killing effect on microbes (Table 2), but weak antioxidant activity (Figure 3B,C). This was different from Cathelicidin-OA1 for it had a strong antioxidant activity [18]. Further, OA-GL21 was observed constantly stable at 4°C which indicated it was easier for transportation and storage at a lower but not higher temperature (Figure 3D).

In summary, chronic wounds caused by ageing and chronic diseases, such as diabetes and uremia, are increasing steadily, but existing wound healing drugs such as small molecular compounds derived from plants and EGFs are difficult to produce, transport, and store. Moreover, growth factors may exhibit a ‘tumor-causing’ like effect, which greatly limits their clinical application [9]. Thus, the small stable peptide OA-GL21, identified from the skin secretions of *O. andersonii*, effectively shortening the process of wound healing and inducing much less formation of scar, may be a potential biological agent for developing new wound-healing drugs for clinical use on chronic wounds.

## Conclusion

In the present study, we identified a new 21-resiude peptide, OA-GL21, from the skin secretions of *O. andersonii*, and determined its sequence as ‘GLLSGHYGRVVSTQSGHYGRG’. OA-GL21 showed no antimicrobial activity, hemolytic activity, or acute toxicity, but weak antioxidant activity. It was worth noticing that OA-GL21 had a strong wound-healing-promoting activity *in vitro* and *in vivo*. Our study provided a new peptide template for the research of new wound-healing medicines.

## Abbreviations

ABTS: A2,2′-azino-bis(3-ethylbenzothiazoline-6-sulphonic acid)
ACN: acetonitrile
DPPH: A2,2-diphenyl-1-picrylhydrazyl
EGF: epidermal growth factor
HaCaT: human keratinocyte
HSF: human skin fibroblast
i.p.: intraperitoneal
RP-HPLC: reverse-phase HPLC
TFA: trifluoroacetic acid
